# Indoor Multi-Sensor Acquisition System for Projects on Energy Renovation of Buildings

**DOI:** 10.3390/s16060785

**Published:** 2016-05-28

**Authors:** Julia Armesto, Claudio Sánchez-Villanueva, Faustino Patiño-Cambeiro, Faustino Patiño-Barbeito

**Affiliations:** 1 Mining Engineering School, University of Vigo, A Xunqueira, Pontevedra 36005, Spain; 2 Industrial Engineering School, University of Vigo, Rúa Maxwell, Vigo 36310, Spain; csanchez@uvigo.es (C.S.-V.); faustinopc@uvigo.es (F.P.-C.); 3 Industrial Engineering School, University of Vigo, Rúa Conde de Torrecedeira 86, Vigo 36208, Spain; inardesign@uvigo.es

**Keywords:** building rehabilitation, energy efficiency, indoor mapping, SLAM

## Abstract

Energy rehabilitation actions in buildings have become a great economic opportunity for the construction sector. They also constitute a strategic goal in the European Union (EU), given the energy dependence and the compromises with climate change of its member states. About 75% of existing buildings in the EU were built when energy efficiency codes had not been developed. Approximately 75% to 90% of those standing buildings are expected to remain in use in 2050. Significant advances have been achieved in energy analysis, simulation tools, and computer fluid dynamics for building energy evaluation. However, the gap between predictions and real savings might still be improved. Geomatics and computer science disciplines can really help in modelling, inspection, and diagnosis procedures. This paper presents a multi-sensor acquisition system capable of automatically and simultaneously capturing the three-dimensional geometric information, thermographic, optical, and panoramic images, ambient temperature map, relative humidity map, and light level map. The system integrates a navigation system based on a Simultaneous Localization and Mapping (SLAM) approach that allows georeferencing every data to its position in the building. The described equipment optimizes the energy inspection and diagnosis steps and facilitates the energy modelling of the building.

## 1. Introduction

Energy building rehabilitation has become an opportunity for the European construction sector, which has suffered strongly from the economic crisis of previous years. However, it is also an essential task in several European countries to reduce the energy dependence, since nearly 40% of the total energy consumption is due to existing buildings (Directive 2010/31/EU) [1]. About 75% of buildings in European Union were built during the absence of energy efficiency codes. According to the Energy Efficiency Financial Institutions Group [2], depending on the European Commission and United Nations Environment Program Finance Initiative, 75%–90% of those standing buildings are expected to remain in use in 2050. Buildings represent the greatest potential to save energy in EU. Further, the recent 2015 Paris Climate Conference concluded in the agreement of keeping global warming below 2 °C. To this end, the reduction of greenhouse gases emissions and, consequently, the increase of energy efficiency, is urgently needed. In this frame, energy rehabilitation of existing buildings has become a strategic goal. The challenge in reducing energy use in buildings lays in improving diagnosis, increasing the effectiveness of rehabilitations, and providing energy savings in terms of costs and emissions in order to justify the investments to be made. New methodologies to evaluate building performances and to reduce the gap between predicted and achieved savings are needed.

In building rehabilitation different disciplines converge; thermodynamics, and computer fluid dynamics are involved in energy modelling and simulation, construction, and engineering are needed for defining models, the interpretation of simulation results, and the implementation of actions. However, advances in geosciences and robotics in the last decades can be quite useful for the building rehabilitation chain, either for the capturing of information, the diagnosis, or the quality control step.

Traditionally a detailed study of a building before rehabilitation consists of the collection of CAD files or drawings of the original project and a reforms log, thermographic inspection to document thermal bridges, moisture, and infiltration, recognition of the materials of the construction, identification of features, and intensities of use and building loads. The construction of an energy model that compiles this information, simulates the energy behavior, and evaluates energy efficiency begins with the transposition of the available 2D CAD files or drawings of the building to a 3D space. Several difficulties usually arise in this process given the variety of formats in which the information is stored (plans on paper, obsolete digital formats, new digital formats, *etc.*), the dispersion of the information (administration, owner, *etc.*), and other existing variations between the executed project and the original one. In practice, energy models are commonly based on deductions derived from the building construction date and the prior knowledge of the materials and techniques used in that period.

Software solutions can be found that allow creating 3D models for energy simulation purposes: direct sketching the model or using original CAD files or drawings, or adapting the designed geometry to the thermal modelling spaces of the building. The building modelling step usually consumes considerable time in the process of energy evaluation: it can represent up to 70% of the entire time. The 3D modelling is frequently a manual process affected by the subjectivity of the technician, with the result that different technicians can generate different energy models from the same plans. Finally, when CAD files or drawings are not available, or the building has undergone a substantial reform, it is necessary to accomplish the survey of the building. These surveys are usually carried out through traditional techniques, which can be precise but limited and highly simplified given the high costs involved.

In 3D modelling laser technologies are a competitive tool nowadays. Stationary terrestrial scanning systems consist in laser heads that emit thousands of pulses per second so that they can measure millions of 3D points of a built environment in a few seconds, in a flexible and very precise way. From the 1990s until now, terrestrial laser scanners (TLS) have been widely applied in several fields: cultural heritage documentation [3,4,5], forest surveys [6,7], landslide monitoring [8,9], urban planning [10,11], and building modelling [12]. In energy modelling applications, TLS can be successfully used in façade modelling. However, these are line-of-sight instruments and they have a limited measurement range, therefore multiple scans from different viewpoints are needed in order to ensure the full coverage of a complex environment, like indoor scenarios.

Mobile laser scanning systems have been developed that allow the integrated moving of platforms and laser scanning heads. Inertial systems and GPS units are used to fix the laser profiles in the global reference system. There are several commercial equipment that are used in roads, bridge, and tunnel inspection, work control, and large open spaces surveys: Riegl VMX 450© (Horn, Austria), Optech Lynx SG© (Ontario, ON, Canada), Trimble MX8© (Sunnyvale, CA, USA) and Topcon IPS3© (Tokyo, Japan) are some examples. Nevertheless, these mobile mapping systems are limited to areas where a GPS signal is available. Indoor scenarios, thus, require different solutions. Position and attitude might be determined without a high-performance global positioning system (GPS) or an inertial measurement unit (IMU). Vision navigation via real-time image processing of data gathered from imaging sensors deals with this task. DeSouza and Kak [13] described three different approaches: map-based, map building-based, and mapless navigation. The map-based approach provides the mobile platform with a map of the environment, while the mapless approach is a relative navigation technology aimed at understanding surroundings and a local environment without prior description of it. This approach is useful for indoor navigation and inspection purposes since a campaign can be easily accomplished without the need of explorations or maps of the area to be analyzed. In mapless navigation, sequences of images are used to determine the relative position and orientation information of the imaging sensor. The recently-developed Simultaneous Localization And Mapping (acronym: SLAM) technique uses imaging sensors to extract the navigation information and to reconstruct the 3D environment data. It mainly consists in building the map by themselves. This is called the map building-based approach. SLAM is used in self-driving cars [14], unmanned aerial vehicles [15], autonomous underwater vehicles [16], or domestic robots.

Research in SLAM has achieved impressive results in recent years in the field of indoor mapping. Borrmann *et al.* (2008) [17] distinguish four categories of SLAM approaches using laser scanners: (a) A planar localization method and bidimensional mapping with a 2D laser scanner; (b) a planar localization method combined with a 3D scanner that capture the surrounding geometry (TIMMS©, Sunnyvale, USA, and i-MMS© systems, Louverné, France); (c) 3D positioning combined with a 2D laser scanner; an additional sensor is required for the pose estimation, such as an IMU or visual odometry. The Zebedee© scanner (Canberra, Australia) [18] combines an IMU with a 2D laser scanner; and (d) 3D positioning combined with a 3D imaging device. Typically, in the last option, 3D scans are obtained at many close locations with a stop-and-go approach using a terrestrial laser scanner [17], or with depth cameras like Kinect, that instantaneously captures a 3D point cloud [19]. Recently, Lauterbach *et al.* [20] have presented a backpack solution.

Some of the indoor mapping solutions follow the multi-sensor approach by integrating optical cameras. Liu *et al.* [21] developed an experimental prototype which consists of four 2D laser scanners, two cameras, and an inertial sensor in a backpack system. TIMMS© and i-MMS© systems integrate the laser heads and IMU, but also a Ladybug panoramic camera which provides a realistic textured model.

In the last decade some authors explored the integration of geometric information in conjunction with infrared thermal images (IRT). Thermography is essential in building inspections because it enables the location of thermal bridges and the detection of air and moisture infiltrations without physical contact. The information is stored in an image format called thermogram, where the digital value of every pixel is proportional to the temperature value of the surface. The combination of surveying techniques with IRT allows the detection of thermal features and quantify the area affected by them. Different approaches have been described in the References, with image-to-point-cloud-registration being the most common. A point cloud is usually gathered with a terrestrial laser scanner [22,23].

This paper describes an integrated system that allows obtaining the geometric, thermographic, and comfort information inside the building in order to automate acquisition and processing. Light levels, temperature, and humidity are measured with the subsequent goal of checking the compliance with the current legislation on health care, comfort, and work safety under which the building was constructed. Section 2 describes the developed device and subsequent acquisition data processing methodology. Section 3 contains the inspection and evaluation of an existing building. Section 4 shows the validation of the system. Finally, conclusions are summarized.

## 2. Energy Inspection and Modelling Methodology

### 2.1. Platform Description and Sensor

The Indoor Multi-sensor Acquisition System (IMAS) presented in this paper consists of a wheeled platform equipped with two 2D laser heads, RGB cameras, thermographic camera, thermohygrometer, and luxmeter. One of the laser scanning sensors is foreseen to obtain the building map and the navigation information, and the other one to the 3D environment reconstruction. The thermographic and optical images, and the geometric and comfort data are synchronized and automatically linked to trajectory positions, so that they are georeferenced in the building in terms of a relative positioning system.

**Figure 1:**
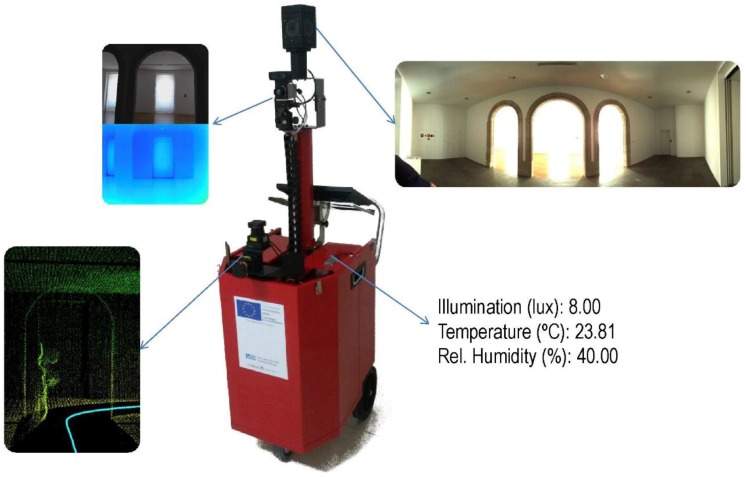
Indoor Multi-sensor Acquisition System.

The moving platform has been designed and built *ad hoc* for building inspection. It consists of a metallic box mounted on three wheels, with a telescopic arm which holds the cameras in the upper part, a support for a touch screen in the front and a rigid mounting for laser heads in the back. The closed box holds the battery, control computer, and auxiliary devices. Mobile parts allow two configurations: transport (folded) and working (unfolded). In the former, the moving parts are spread in a suitable position for measurement, minimizing occlusions between sensors (Figure 1).

Technical characteristics of the sensors are detailed in Table 1. The laser unit involved in the 3D reconstruction is mounted in the rigid support with the Z-axis aligned with the direction of motion, so that vertical laser profiles are obtained and the measured coordinates for each point are (y, z). The 90° blind sector of the laser head is oriented to the floor in order to minimize missing data from walls and ceilings of the inspected rooms. The laser unit involved in the trajectory computation is mounted in horizontal position, with its Z-axis aligned with the Z-axis of the platform, so that horizontal profiles are obtained and the coordinates measured for each point are (x, y). In this case the blind 90° sector is oriented to the operator.

The thermographic camera is placed in the telescopic arm aimed in the direction of motion. It is mounted on a ball joint to pinpoint any incidence in detail by pointing the camera in the needed direction. The 360 RGB camera is mounted in the upper part of the telescopic arm. Indoor comfort conditions are disclosed by the luxmeter and thermohygrometer.

### 2.2. Data Acquisition

**Figure 2:**
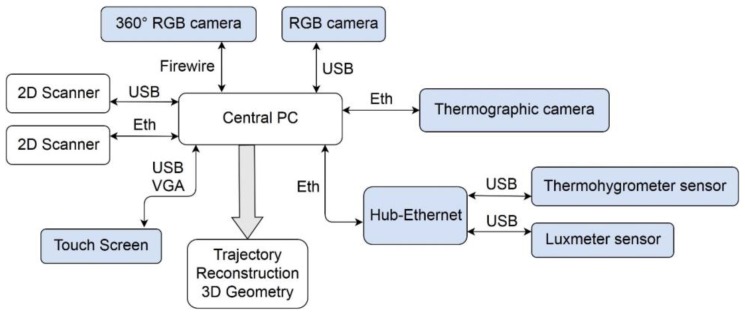
Connections scheme of the IMAS components.

The IMAS system is pushed and conducted by one operator through the thermal spaces of the building for inspection at normal human walking speed (0.8–1.1 m/s). The multi-sensor acquisition is performed automatically along the inspection route, the system being managed by a control computer (see Figure 2). Only the panoramic images are gathered by manually-operated single-shots. A log master file is generated where a timestamp in µs is associated to every data file for further linking. Stops and additional acquisitions can be made in the presence of singularities or incidences in the room; for instance, thermal bridges or damp patches on walls that can be thoroughly registered by aiming the thermographic camera in the appropriate direction.

The overall time for data acquisition in the energy inspection of a building will depend on the size of the building, but also on its geometrical configuration (open spaces, size and complexity of rooms, furniture, *etc.*) and singularities that might be additionally documented. The quality of the data in terms of resolution in the 3D point cloud is not a function of the acquiring time or speed for collecting the data. However, there is a key factor that affects the resulting data. The higher the acquisition speed, the faster the world changes around the system, and the Hector SLAM algorithm can fail in the successive scan matching. An absolute value cannot be provided since this task also depends on the geometrical characteristics of the surrounding environment.

The time lapse for IMAS data acquisition can be configured by the user as linear or angular intervals in the forward inspection path. Under normal operating conditions, IMAS positions are computed every 20 ms. For 0.8 m/s operation speed, the estimated trajectory positions are distanced 17 mm. The accumulated trajectory is calculated in real-time. The closest trajectory positions to the predefined lapse intervals are selected, the corresponding time stamp is read and, finally, the images and vertical laser profiles with the closest time stamp are saved. This way the sensor synchronization is guaranteed. Energy modelling purposes do not require dense 3D point clouds, or redundant optical images, thermographic images, and ambient data, so the predefined lapse for data acquisition is expected to be always much higher than the distance between estimated trajectory positions.

### 2.3. Indoor Navigation System

The inspection trajectory reconstruction is essential for the georeferencing of the data and the *ex situ* inspection, but also for the 3D reconstruction of the indoor environment of the building. For the data processing and the acquisition management we used the ROS (Robotic Operating System). ROS contains numerous open source libraries. In ROS, each step constitutes an independent process called a node. The inputs and outputs of nodes are called topics.

For the trajectory reconstruction we used the Hector SLAM algorithm [24]. The process consists in building an occupancy grid map of the surrounding environment in real-time using the horizontal laser scan. The scan matching algorithm for aligning successive scans is based on the Gauss-Newton approach [25]. Important advantages of this SLAM technique are that it requires low computation and is able to avoid major changes in the map during operation [26]. However, the compatibility of the IMAS acquisition with the normal operation of the building will depend on the number of people moving around.

**Figure 3:**
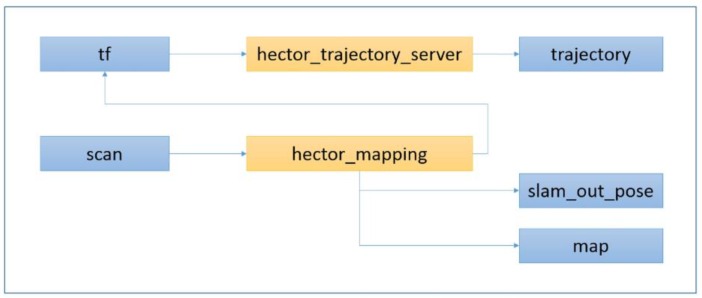
Hector SLAM scheme: nodes (yellow) and topics (blue).

**Figure 4:**
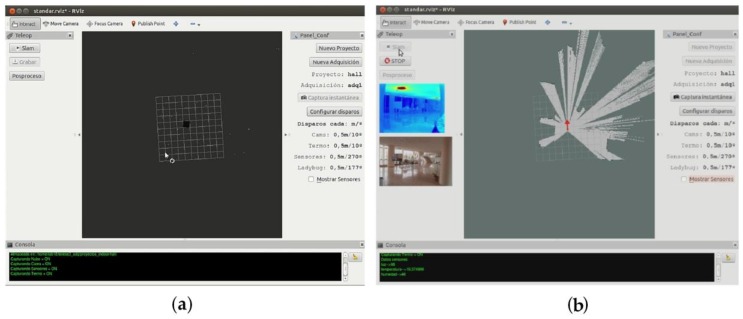
Screen captures of the software developed for controlling data acquisition at: (**a**) standby mode; and (**b**) working mode.

The Hector SLAM system is designed with six degrees of freedom. The indoor floors of buildings are flat in most cases, so roll and pitch are assumed to be 0, and the Z coordinate is considered as a constant. The system is designed to avoid odometry data, purely relying on fast scan-matching at the full LiDAR update rate. The main nodes and topics that we used are shown in Figure 3. The output of the Hector SLAM system is composed of the trajectory of the IMAS platform and the GeoTIFF file that contains the horizontal scanned area. A software application in ROS has been developed (see Figure 4) that allows a real-time visualization of the scanned area, the current position of the platform, previous trajectory positions, thermographic and optical images, as well as the comfort data. The interface also includes a configuration panel to determine the acquisition intervals.

### 2.4. 3D Reconstruction

**Figure 5:**
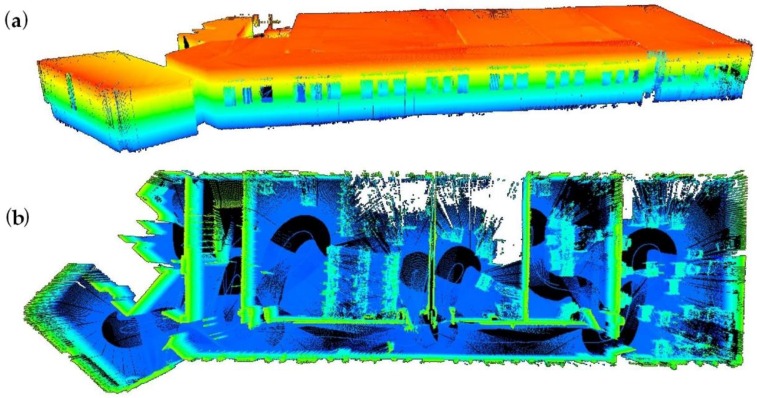
Building 3D point cloud acquired with the IMAS: (**a**) walls and floors in the perspective view; and (**b**) the whole point cloud in perspective.

The generation of the 3D point cloud is achieved by placing each 2D laser profile at the corresponding path position in the moment of the acquisition. Consecutive profiles are projected to their respective positions, considering the direction of motion as the third dimension of the point cloud. Figure 5 shows some examples of 3D point clouds acquired with the multi-sensor acquisition system.

### 2.5. Ex Situ Inspection

**Figure 6:**
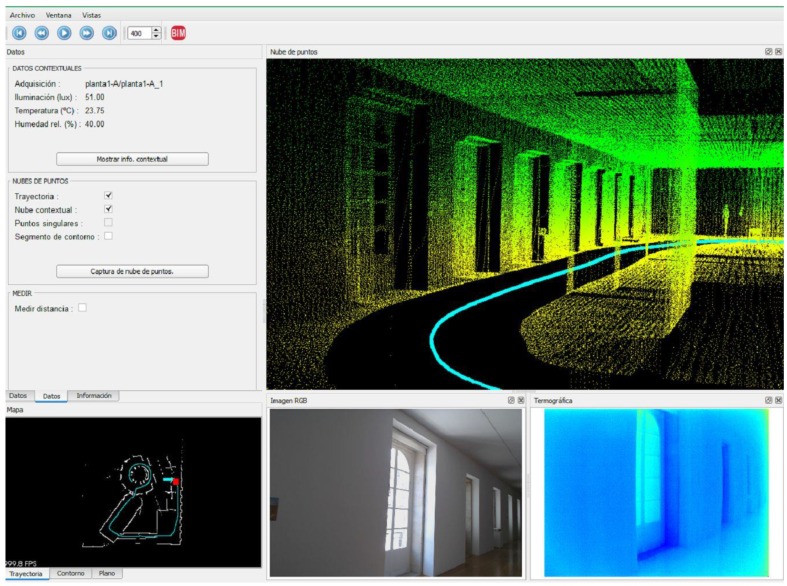
Software interface for virtual immersive navigation and *ex situ* data analysis.

**Figure 7:**
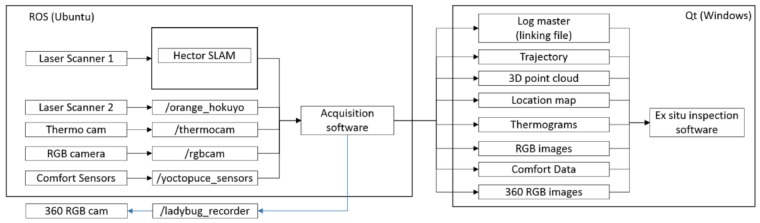
Overall software architecture of the IMAS system. The blue arrow represents the panoramic camera triggering.

All the acquired data can be analyzed *ex situ*. A specific software application has been developed based on the cross-platform application framework Qt to load and show the synchronized data on the office computer (see Figure 6). The interface has been designed to allow a virtual immersive navigation through the data after reconstructing the user’s point of view at the moment of the acquisition. The point cloud is reprojected into a linear perspective view from every operator position along the inspection path. Since all of the data were synchronized and linked through timestamps, corresponding thermographs, images, and comfort data are shown simultaneously. Further, a horizontal section of the point cloud is obtained and shown as a plan view, which it is used as a localization map that provides the position of the user in the building. Travelling forward and backward along the inspection path is allowed, since the gathered data are updated as a video sequence. A floating mark showing the current position is updated accordingly to the virtual navigation at the location map. The system allows to generate thematic maps containing the spatial pattern of the illumination, temperature, and relative humidity data. Figure 7 shows the general architecture of the software system.

### 2.6. Energy Modelling and Evaluation

Once the data are acquired, they can be stored and transferred to the office, where an energy modelling and assessment can be carried out. Firstly, the energy evaluation technician can perform a virtual navigation through the data in an immersive environment. In this way the interpretation of results is optimized and thermal leakages can emerge. Therefore, thermal incidences can be detected in the thermographic sequence and visual images permit the interpretation. If they were considered as thermal bridges, they could be immediately measured in the 3D point cloud. The availability of temperature and humidity maps facilitates the detection of insulation problems in outer walls or windows, whilst the illumination map allows to evaluate the working conditions. Further, the global 3D point cloud can be automatically generated once the acquisition has concluded. This 3D geometry can be transferred to an energy evaluation software, where the orientation, ambient conditions, climate data, and HVAC systems complete the definition of the energy model. Then, the energy simulation can be performed.

The greater the accuracy of the collected data is, the more precisely the virtual model captures the physical parameters of the indoor environment. Hence, the energy modelling will provide us a reliable result of the energy performance of the entire building. Consequently, the simulation of renovation solutions will be also more reliable. The IMAS could be used to evaluate the effectiveness of renovation actions in comfort variables, thermal bridges, air infiltrations, *etc.*, if the inspection campaign is accomplished after any renovation intervention.

## 3. Energy Modelling and Evaluation: A Case Study

The theoretical background explained in the present study was validated by using the IMAS in the process of obtaining the energy certification of an educational building. The Forestry Engineering Technical School (Pontevedra, Spain) was considered. The building dates from the early 1990s and consists of four storeys. While, on the outside, no signs of relevant reforms are perceived, inside the building the layout and uses of the rooms have been modified from the original configuration.

The modelling of the building was carried out following the simplified procedure described in the regulation of the Energy Efficiency Certificate established by the Spanish applicable standard. This method requires the determination of a set of general parameters of the building that include the geographical location, the geometric characteristics of the volumes to be heated, the mass of the interior walls (to calculate the effect of indoor thermal mass), the types of use of the building, the existing regulations at the time the building was constructed, hot water consumption, thermal bridges, enclosure materials, heating and air conditioning installations, air renewal, and lighting.

**Figure 8:**
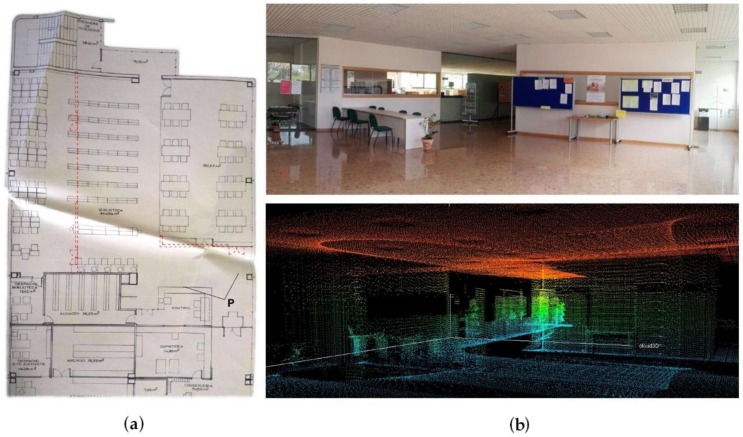
Deviations revealed after the inspection: (**a**) plan view; and (**b**) the as-built environment.

IMAS was used to reliably gather the data regarding the geometry and thermal bridges inside the building. The surface of the building in the study case is 2520 m^2^ per floor; the time to acquire the data was less than two hours in every floor, since rooms are quite large and open spaces prevail. The geometric model was created and then compared with the information contained in the original drawings of the buildings. Since the model obtained through the inspection system represents the real geometry (with a certain margin of error), significant differences were detected due to changes in the use of the building that were not included in the existing documentation (see Figure 8). For this reason, special attention was paid to the geometric data collected during the inspection.

**Figure 9:**
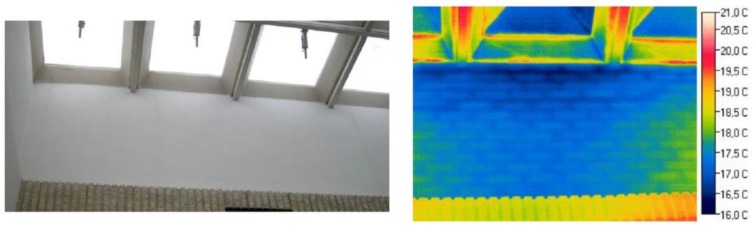
Indoor thermogram and RGB image of a skylight revealing air infiltration and thermal bridges.

Secondly, the thermographic data collected were compared with the available information about the materials that constitute the enclosure walls, extracted from the building technical documentation as well as from the experience, knowledge and reasoning skills of the technician responsible for the energy certification. Next, the goal was to detect divergences between these sources of information, modifications not reflected and thermal bridges such as skylights, stiffeners of prefabricated structural elements, *etc.* (see Figure 9).

Finally, the HVAC systems were assessed in terms of their efficiency during the use of the building. The real efficiency was estimated from the theoretical efficiency given in the technical specifications and the estimation of the efficiency loss corresponding to the maintenance applied.

Once all of these required data were collected, the Energy Rating of the building could be calculated. The parameters obtained were an energy demand of heating and of cooling of 58.3 and 8.69 kWh/m^2^/year, respectively (energy rating E); primary energy consumption of 204.52 kWh/m^2^/year (energy rating C); finally, obtaining the global indicator of 52.12 kg CO_2_/m^2^/year of gas emissions, which corresponds to a global Energy Efficiency Rate of class D. In conclusion, it is necessary to improve the building envelope in order to reduce thermal bridges and high thermal transmittance through the openings, with the possibility of also improving the energy systems.

## 4. IMAS Quality Analysis

The key task in the IMAS system is the quality of the reconstructed trajectory, since all data are referred to trajectory positions. The trajectory can be evaluated by comparing the 3D point cloud generated by the IMAS with a point cloud obtained with a FARO Focus3D (Lake Mary, FL, USA) terrestrial laser scanner. This equipment is a phase-based system with an accuracy of ±2.2 mm at 25 m. First, 3D distances of significant features are measured in both point clouds: length, height, and width of corridors, rooms, and doors, and distances between geometrical features. Absolute and relative errors for some examples are shown in Table 2, and general statistics in Table 3. The width and height dimensions are aligned with the scan vertical profile in most cases, so the errors in this cases are due to the laser head characteristics and the visual error in the selection of the features points. The lengths are dependent on the visual error, as well, but also on the quality of the 3D point cloud reconstruction. In every case, relative errors have been kept below 1%.

Second, the accuracy of the IMAS point cloud is estimated through a point to point comparison according to the methodology proposed by Aguilar and Mills [27]. Distances between a random sample of 10,000 points in the IMAS point cloud and the closest ones in the ground truth point cloud are calculated. Previously, a manual filtering is applied in order to remove noisy points coming from people, furniture, exterior buildings visible through windows, and other objects not related with the building under study. Then, both point clouds are referred to the same coordinate system through affine transformation consisting of applying translation and rotation. Finally, the sample of points is selected and distances between these and the closest ones in the ground truth point cloud are calculated. In the case discussed here, the mean of distances is 0.0953 m; the standard deviation is 0.0928 m.

**Figure 10:**
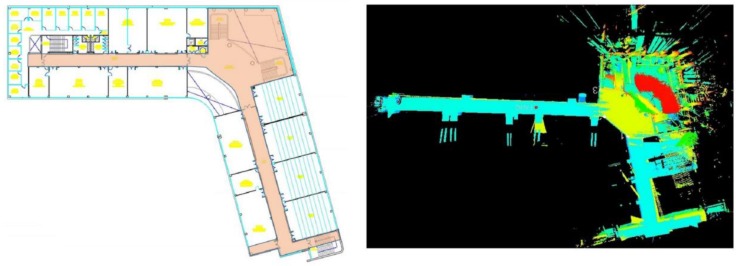
Plan view of the scanned area and overlapping point clouds for drift evaluation.

Finally, the drift of the IMAS is evaluated. The drift is due to the accumulation of the sensor errors during the path reconstruction process performed by the SLAM algorithm. It becomes significant as the length of scanned areas increases. To evaluate this error, significant corresponding points in both point clouds are selected among the features captured at the end of a measuring path. Distances between pairs are obtained for inspection paths of 31 m and 126 m length. Some examples are shown in Table 4. In every case, the maximum distances are below 3 m. The drift can be appreciated visually when IMAS and reference point clouds are superimposed (see Figure 10).

## 5. Conclusions

This paper presents the design and development of an indoor multi-sensor acquisition system that allows performing a full 3D modelling and energy inspection of the building quickly and in a single session. The system is equipped with laser scanners, thermographic and optical cameras, 360° RGB camera, a thermohygrometer, and a luxmeter. All of the sensors are triggered simultaneously and a time stamp is assigned to each sensor file. Consequently, an optimal framework for *ex situ* inspection is provided: the inspector can perform a virtual navigation through the data in an immersive frame, where all the acquired data are shown and sequenced as a video. This approach facilitates the interpretation of the results: thermal incidences can be detected in the thermographic sequence, visual images allow their correct interpretation, and the 3D point cloud permits measuring the dimensions of the elements involved in the thermal bridges.

Moreover, obtaining a 3D point cloud in an automated way substantially accelerates the 3D reconstruction process for further energy simulation. The quality analysis of the point cloud has revealed satisfactory results for energy evaluation purposes. Errors in the measuring of features are below 1%, the platform drift is below 3%, and the point-to-point comparison resulted in a standard deviation under 10 cm.

The Energy Efficiency Rate described in the European regulation is a parameter intended to quantify the potential energy savings of a building. Gathering reliable and updated information about the technical details of the building and the energy systems installed, allows the minimization of the error in the calculation of this parameter, apart from allowing it to identify and evaluate thermal losses with a satisfactory accuracy. In consequence, the information provided by the IMAS can highlight measures to be taken in pursuance of reducing the energy consumption and, thus, CO_2_ emissions.

At present, work is being carried out to provide the IMAS platform with sensors that are able to measure gases generated by the heating boiler, in conjunction with sensors used to monitor the efficiency of the energy installations. These extensions would allow the documentation of all of the variables involved in the energy certification process.

## Figures and Tables

**Table 1:** Technical characteristics of the sensors installed on the IMAS.

**Hokuyo UTM-30LX**	**XenicsGobi-640**	**Logitech C920**
Scanning principle: time of flight	Thermal sensor: Uncooled Microbolometer Array	Optical sensor: CMOS
Measurement range: 30 m	Spectral range: LWIR 8–14 µm	Sensor size: 2034 × 1536 pix
Measurement angle: 270°	Temperature range: −40–60 °C	FOV: 78°
Angular resolution: 0.25°	Array size: 640 × 480 pix	
Measuring rate: 0.04 ms	Frame rate: 50 Hz	
**Ladybug 3**	**Yocto-Light**	**Yocto-Light**
Optical sensor: CCD	Type of sensor: Luxmeter	Type of sensor: Thermohygrometer
Size: 1600 × 1200 pix (6)	Measuring range: 3 to 65,000 lux	Measuring range: −40–125 °C, 0%–100% RH
	Accuracy: 5%	Accuracy: 0.3 °C, 4% RH

**Table 2:** Quality evaluation of the 3D point cloud obtained with the IMAS: geometric parameters of characteristic features.

	IMAS (m)	FARO (m)	ε (m)	ε_r_ (%)
Corridor length	31.577	31.527	0.05	0.16
Corridor height	3.009	3.000	0.009	0.30
Corridor width	3.022	3.018	0.004	0.13
Hall length	12.651	12.628	0.023	0.18
Small corridor length	14.492	14.427	0.065	0.45
Room length	14.232	14.12	0.112	0.79

**Table 3:** Quality evaluation of the 3D point cloud obtained with the IMAS: General statistics.

	ε (m)	ε_r_ (%)
Mean	0.017	0.29
Standard deviation	0.040	0.49
RMSE	0.023	0.03
Maximum	0.112	0.98

**Table 4:** Drift evaluation of IMAS; inspection path: 31 m.

	Point 1	Point 2	Point 3	Point 4	Point 5	Point 6
ε (m)	0.113	0.098	0.082	0.033	0.027	0.140

## Abbreviations

The following abbreviations are used in this manuscript:

HVAC:	Heating, Ventilating and Air Conditioning
TLS:	Terrestrial Laser Scanners
GPS:	Global Positioning System
IMU:	Inertial Measurement Unit
SLAM:	Simultaneous Localization and Mapping
IRT:	Infrared Thermal Images
IMAS:	Indoor Multisensor Acquisition System
ROS:	Robotic Operating System
LiDAR:	Light Detection and Ranging
